# Agonism of GPR120 prevented IL-1β-induced reduction of extracellular matrix through SOX-9

**DOI:** 10.18632/aging.103375

**Published:** 2020-06-24

**Authors:** Zhixian Xu, Tie Ke, Yongfa Zhang, Chaofeng Fu, Wubing He

**Affiliations:** 1 Department of Emergency Surgery, Shengli Clinical Medical College of Fujian Medical University, Fujian Provincial Hospital, Fuzhou 350001, Fujian, P.R. China

**Keywords:** osteoarthritis, GPR120, GW9508, IL-1β, cartilage matrix

## Abstract

Osteoarthritis (OA) is a whole-joint disease with extremely high prevalence. In all treatment approaches of OA, blocking the degradation of the cartilage extracellular matrix is an important treatment. In OA, overexpression of derivative enzymes leads to excessive catabolism and reduced synthesis of cartilage including type II collagen and aggrecan, which results in irreversible destruction of the joint. SOX9 is a transcription factor that regulates the synthesis of type II collagen and aggrecan and is significantly downregulated in OA. GPR120 has been reported to affect the pathophysiology of OA. In this study, we used the GPR120 agonist GW9508 and TUG891 in ATDC5 chondrocytes exposed to interleukin (IL)-1β to investigate the involvement of GPR120 in SOX9-mediated expression of type II collagen and aggrecan. Our findings show that agonism of GPR120 can reduce inflammation by inhibiting the expression of IL-6 and IL-8 induced by IL-1β. We also show that GW9508 and TUG891 rescue the expression of type II collagen and aggrecan by preventing the reduction of SOX9 expression. Additionally, we demonstrate that the effects of GW9508 on SOX9 expression are mediated through CREB and that GPR120 is indeed required for this effect. Thus, agonism of GPR120 by GW9508 might be a potential therapeutic strategy to halt or prevent cartilage degradation.

## INTRODUCTION

Osteoarthritis (OA) is a common debilitating joint disease most often observed among the elderly. Multifarious factors contribute to an increased risk of OA, such as injury, obesity, genetics, diet, and age, among others. Of these, age is considered the primary risk factor. Therefore, as the average age of the global population increases, so shall the incidence of this painful and costly disease [1, 2]. The main features of OA include excessive catabolism of articular cartilage, inflammation, chondrocyte senescence, oxidative stress, and alterations in the form and function of bone and cartilage tissues [3, 4]. Presently, joint replacement is the go-to treatment for end-stage OA, but especially in elderly patients, surgical intervention can carry additional risks [4]. Therefore, it is of ample importance to develop preventative therapeutic strategies and non-invasive treatment options against OA.

The pathogenesis of OA is complex. Once considered a “wear and tear” disease predominantly associated with cartilage destruction, OA is now recognized as a whole-joint disease involving synovitis, bone remodeling, chondrocyte dysfunction, inflammation, and cartilage destruction, etc. [5]. Despite this multifactorial etiology, preventing or reversing cartilage degradation remains a primary focus of research. Normally, chondrocytes secrete substances to regulate the turnover of type II collagen and aggrecan, the two main components of the articular extracellular matrix (ECM). These include catabolic enzymes such as matrix metalloproteinases as well as signaling molecules that trigger cartilage synthesis [6]. However, OA chondrocytes lose the ability to maintain cartilage homeostasis and enter a state of oxidative stress. Oxidative stress, in turn, triggers the formation of the senescence-associated secretory phenotype (SASP), which results in excessive production of pro-inflammatory cytokines, including interleukin (IL)-1β, IL-6, and IL-8. The SASP also induces functional and phenotypic changes in surrounding cells [7, 8]. IL-1β plays a key role in OA by inducing the expression of degradative enzymes, such as matrix metalloproteinases and aggrecanases, and is often used *in vitro* to simulate OA conditions [9]. SOX9 is a central transcription factor that plays an important role in chondrogenesis by mediating the transcription of type II collagen and aggrecan. SOX9 is expressed during the developmental stage as well as in adults [10, 11]. Thus, overexpression of SOX9 may have therapeutic potential. The cAMP-response element-binding protein (CREB) mediates SOX9 expression by binding to specific sites on the SOX9 proximal promoter region, and mutations at these sites have been shown to reduce SOX9 expression [12].

In recent years, the class of G protein-coupled receptors (GPCRs) has been receiving increasing attention for the ability of its members to modulate specific cellular processes. GPR120, also known as free fatty acid receptor 4 (FFAR4), is an omega-3 fatty acid (ω3-FA) receptor, which has been reported to possess a diversity of physiological functions. GPR120 is widely expressed in various tissue and cell types, including intestinal tissue, adipose tissue, macrophages, and pancreas [13, 14]. GPR120 could also participate in metabolic disorders through regulating the secretion of gut hormone and insulin, food preference, and glucose homeostasis [15]. Interestingly, GPR120 has displayed a robust anti-inflammatory property in macrophages and adipocytes by inhibiting NF-κB activation [16]. Notably, GPR120 has been reported to play an important role in the pathogenesis of OA by regulating inflammation, osteoclast differentiation, and metabolic homeostasis [17]. However, the exact mechanisms involved in GPR120-mediated protection against OA remain incompletely understood. In the present study, we used the selective GPR120 agonists to explore the association between GPR120 activation and SOX9-mediated cartilage preservation. We show that agonism of GPR120 significantly reduced the release of inflammatory cytokines and degradation of articular ECM induced by IL-1β.

## RESULTS

### Expression of GPR120 in ATDC5 chondrocytes

**Figure 1:**
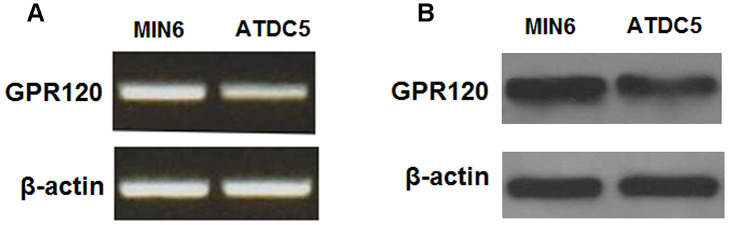
**GPR120 is expressed in ATDC5 chondrocytes.** (**A**) Reverse transcription PCR analysis of mRNA expression of GPR120 with MIN6 cells as a positive control; (**B**) Western blot analysis of protein expression of GPR120 with MIN6 cells as a positive control. Experiments were repeated for three times.

**Figure 2:**
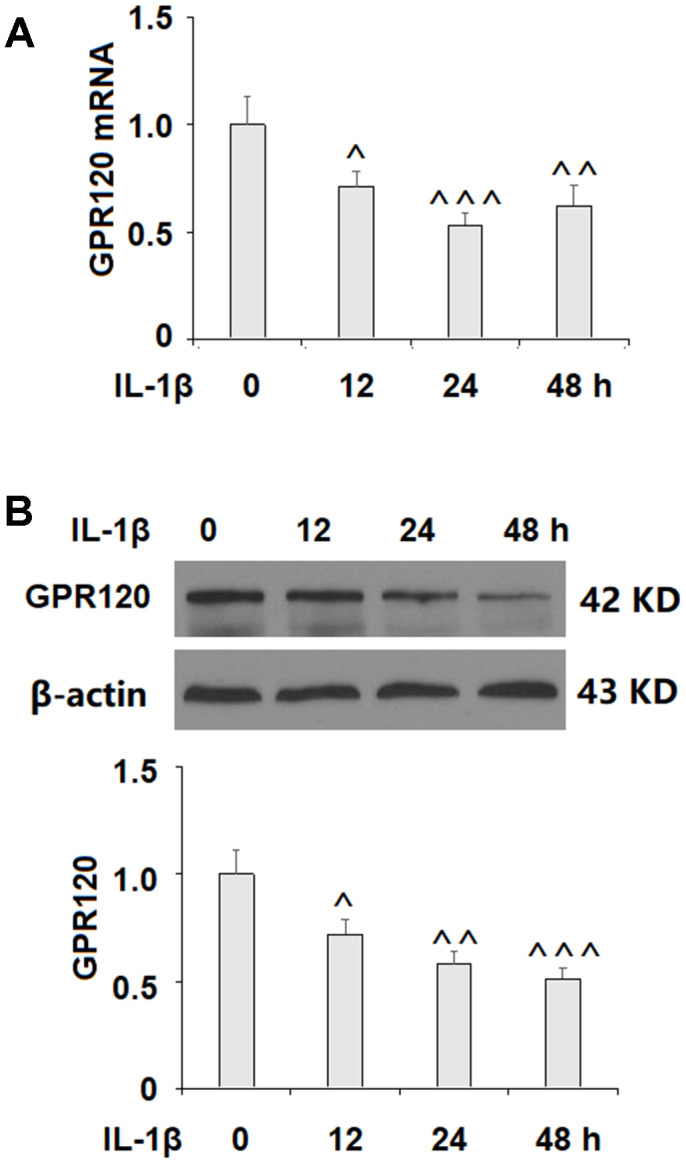
**IL-1β reduced the expression of GPR120 in ATDC5 chondrocytes. Cells were treated with IL-1β (10 ng/ml) for 12, 24, and 48 h.** (**A**) mRNA expression of GPR120; (**B**) Protein expression of GPR120 (^, ^^, ^^^, P<0.01, 0.001, 0.0001 vs. control group, n=4-5).

We began by determining whether GPR120 is expressed in ATDC5 chondrocytes using MIN6 cells as a positive control [18]. As shown in Figure 1, GPR120 is expressed at both the mRNA and protein levels in ATDC5 cells. Next, we assessed the effect of IL-1β on the expression of GPR120 in these cells. The cells were treated with 10 ng/ml IL-1β, and the expression of GPR120 was measured at 12, 24, and 48 h time points. As shown in Figure 2, the mRNA expression of GPR120 was reduced by 29% at 12 h, 47% at 24 h, band 38% at 48 h. The protein expression of GPR120 was reduced by 28%, 42%, and 49% in a time-dependent manner. These results suggest that GPR120 might play a role in mediating the inflammatory response induced by IL-1β.

### Agonism of GPR120 with GW9508 reduces the expression of IL-6 and IL-8

**Figure 3:**
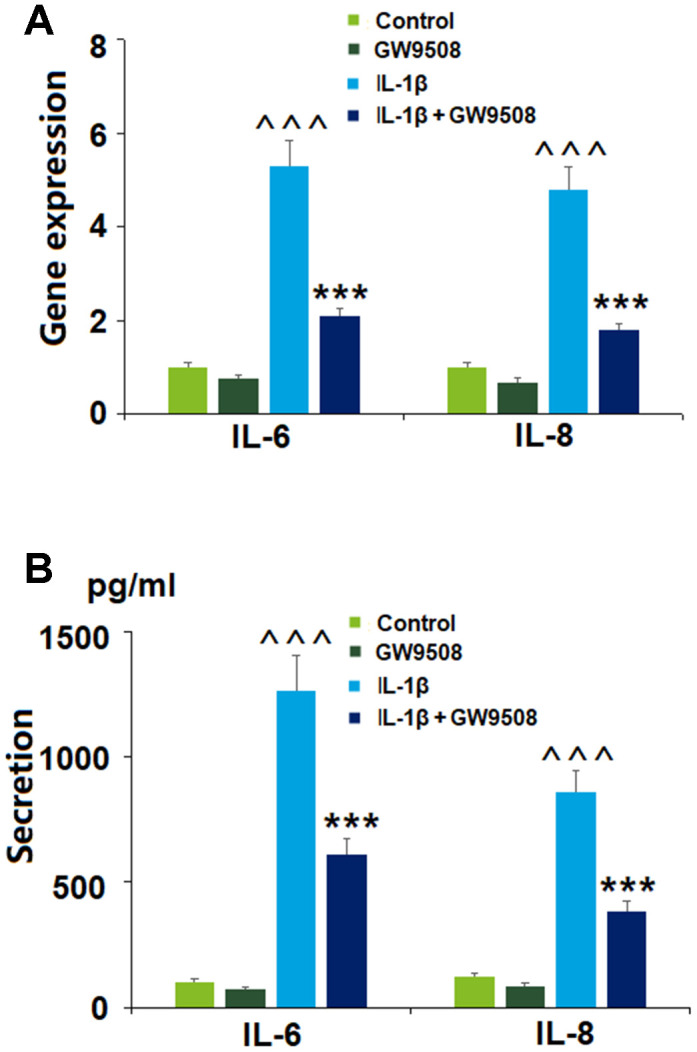
**Treatment with GW9508 reduced IL-1β-induced generation of inflammatory cytokines in ATDC5 chondrocytes.** Cells were treated with IL-1β (10 ng/ml) with or without GW9508 (50 μM) for 24 h. (**A**). mRNA of IL-6 and IL-8; (**B**). Protein secretions of IL-6 and IL-8 (^^^, P<0.0001 vs. control group; ***, P<0.0001 vs. IL-1β treatment group, n=4-5).

Inflammation is well-recognized as a major contributor to the pathogenesis of OA. The cytokine IL-6 has been shown to hinder type II collagen production and promote the expression of catabolic enzymes [19]. IL-8 is an inflammatory chemokine that plays a role in degradative enzyme production and the recruitment and accumulation of immune cells [20]. Here, we explored whether agonism of GPR120 with GW9508 could suppress the expression of IL-6 and IL-8 triggered by IL-1β. As shown in Figure 3, GW9508 was able to significantly limit the IL-1β-induced expression of these two molecules at both the mRNA and protein levels, thereby demonstrating a distinct anti-inflammatory effect.

### Agonism of GPR120 with GW9508 prevented type II collagen and aggrecan reduction

**Figure 4:**
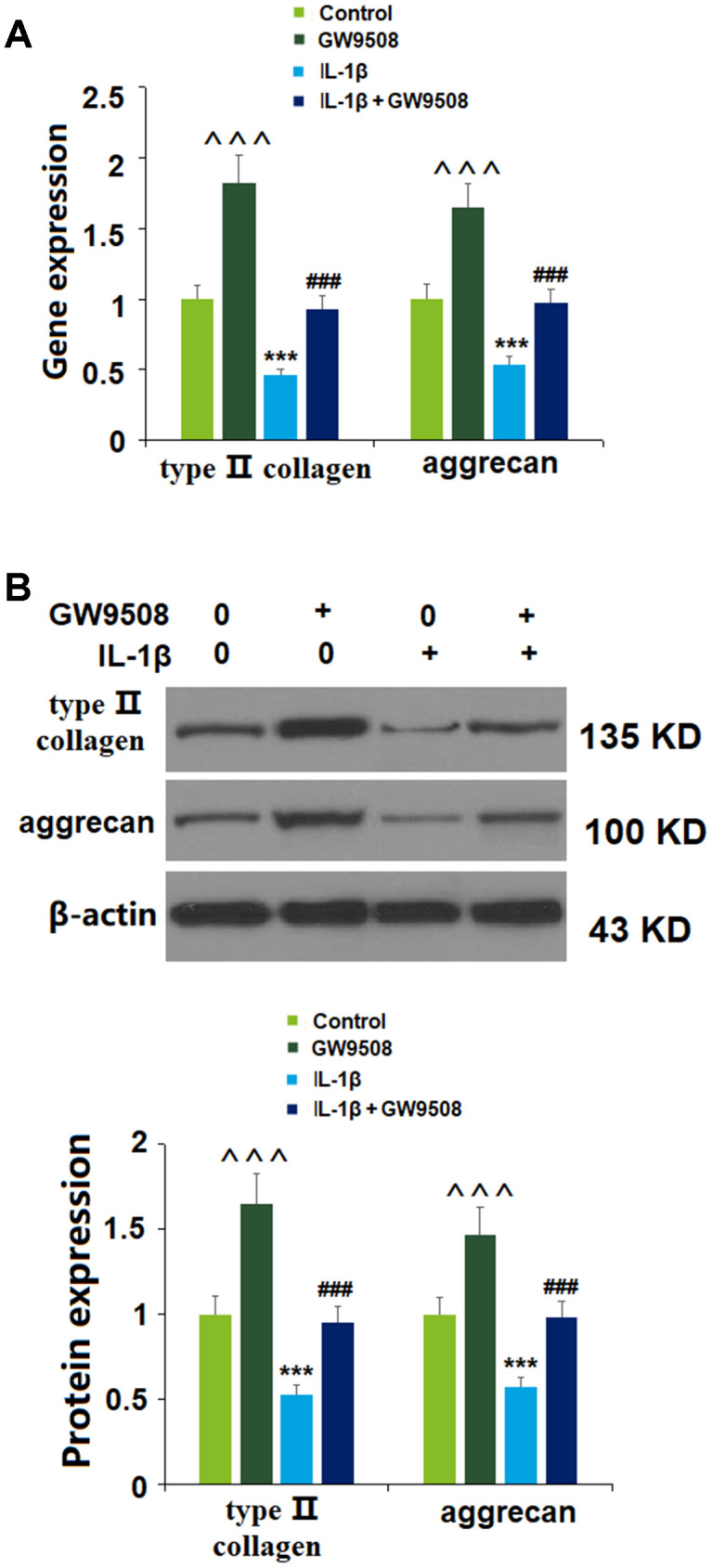
**Treatment with GW9508 prevented IL-1β-induced reduction of type II collagen and aggrecan in ATDC5 chondrocytes.** Cells were treated with IL-1β (10 ng/ml) with or without GW9508 (50 μM) for 24 h. (**A**). mRNA of type II collagen and aggrecan; (**B**). Protein of type II collagen and aggrecan (^^^, ***, P<0.0001 vs. control group; ###, P<0.0001 vs. IL-1β treatment group, n=4-5).

As key components of the articular ECM, preventing the degradation of type II collagen and aggrecan is a vital strategy against the development and progression of OA. We investigated the effects of GPR120 agonism using GW9508 via real-time PCR and western blot analyses. As shown in Figure 4, at both the mRNA and protein levels, IL-1β reduced the expression of type II collagen and aggrecan by about half. Remarkably, the addition of GW9508 almost completely restored these levels to baseline. Notably, treatment with GW9508 alone could increase the expression of type II collagen and aggrecan. Thus, GW9508 shows a significant protective effect against IL-1β-induced degradation of type II collagen and aggrecan in ATDC5 chondrocytes.

### Agonism of GPR120 rescues IL-1β-induced reduced expression of SOX9

**Figure 5:**
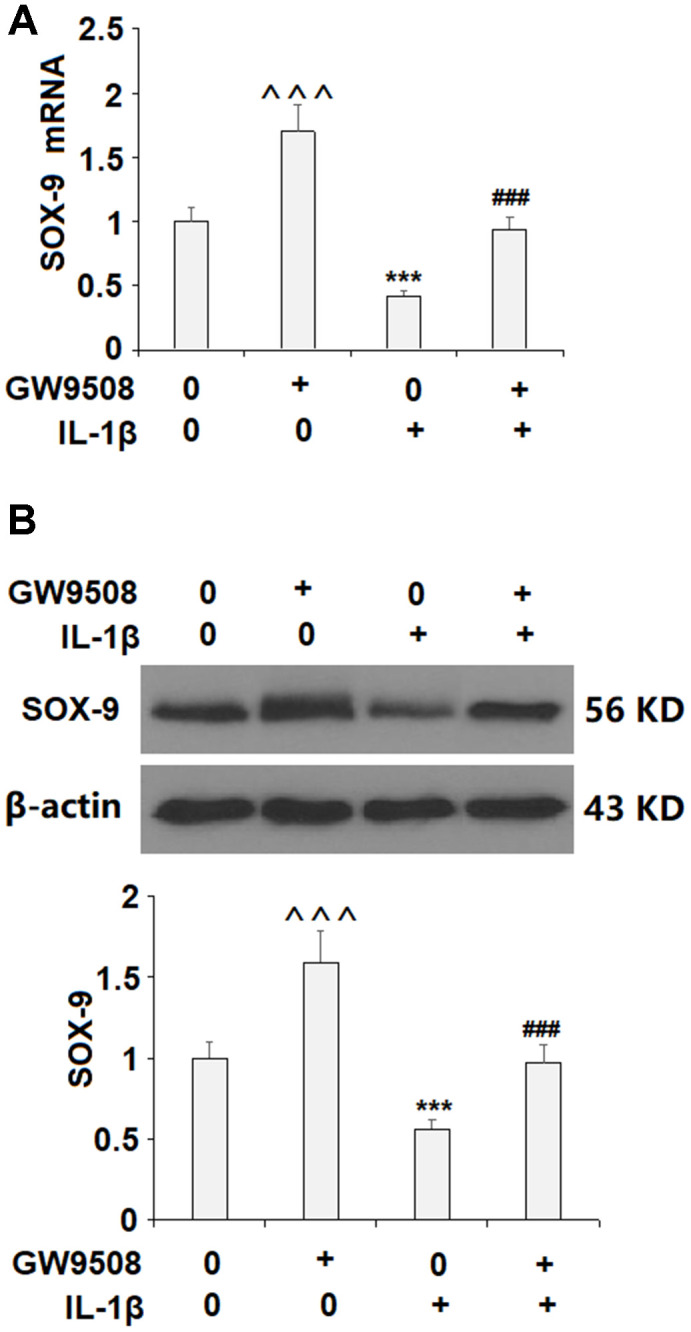
**Treatment with GW9508 restored IL-1β-induced reduction of SOX-9 in ATDC5 chondrocytes.** Cells were treated with IL-1β (10 ng/ml) with or without GW9508 (50 μM) for 24 h. (**A**). mRNA of SOX-9; (**B**). Protein of SOX-9 (^^^, ***, P<0.0001 vs. control group; ###, P<0.0001 vs. IL-1β treatment group, n=4-5).

**Figure 6:**
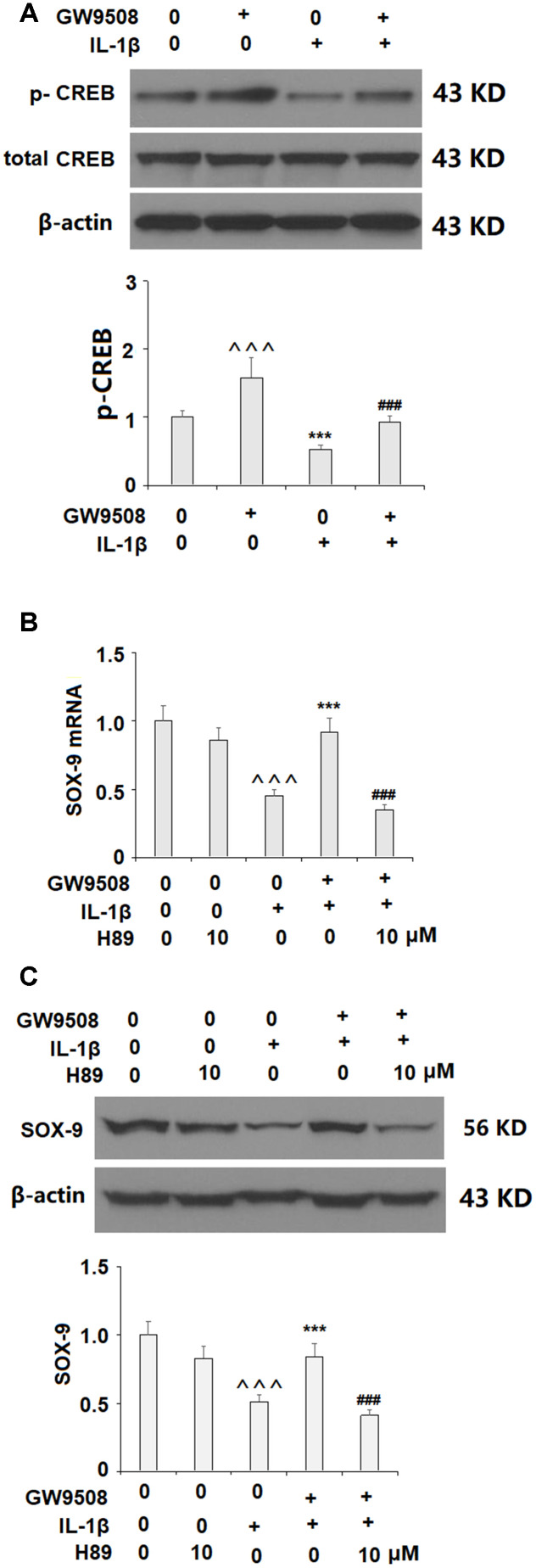
**The effects of GW9508 in increasing SOX-9 expression are mediated by CREB.** (**A**). Cells were treated with IL-1β (10 ng/ml) with or without GW9508 (50 μM) for 24 h. Phosphorylated and total levels of CREB were measured by western blot analysis (^^^, ***, P<0.0001 vs. control group; ###, P<0.0001 vs. IL-1β treatment group); (**B**, **C**). Blockage of CREB with H89 ameliorated the effects of GW9508 in SOX-9 expression. Cells were treated with IL-1β (10 ng/ml) with or without GW9508 (50 μM) or H89 (10μM). mRNA and protein of SOX-9 were measured (^^^, P<0.0001 vs. control group; ***, P< 0.0001 vs. IL-1β treatment group, ###, P<0.0001 vs. IL-1β+ GW9508 group, n=4-5).

**Figure 7:**
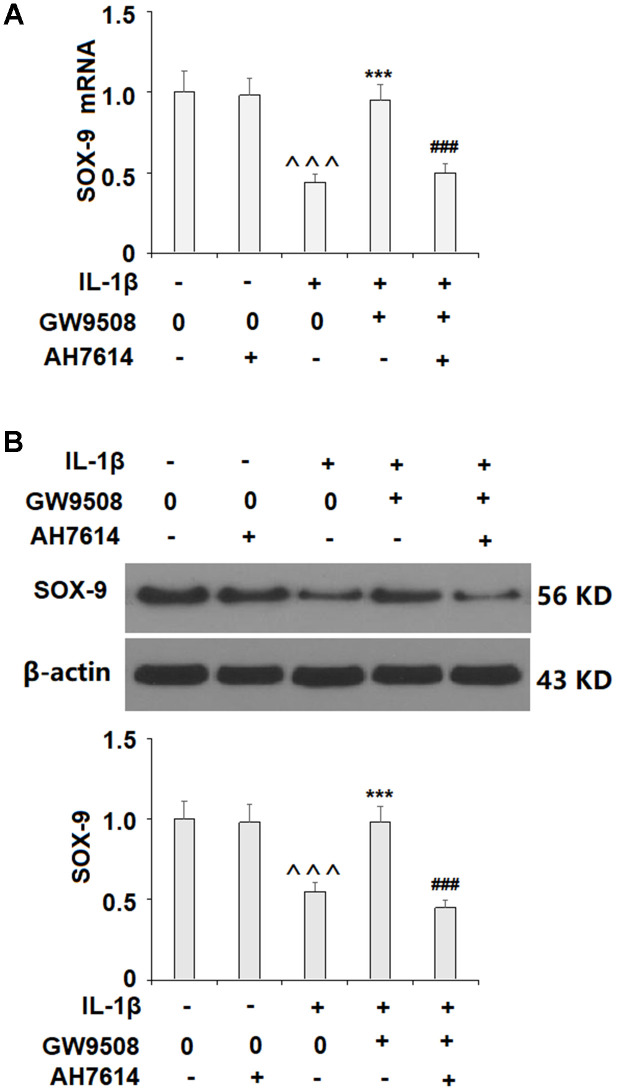
**The effects of GW9508 on abolishing IL-1β-induced decreased SOX9 expression are dependent on GPR120.** Cells were treated with IL-1β (10 ng/ml) with or without GW9508 (50 μM) or the GPR120 antagonist AH7614 (1 μM) for 24 h. (**A**) mRNA of SOX-9; (**B**) Protein of SOX-9 (^^^, P<0.0001 vs. control group; ***, P< 0.0001 vs. IL-1β treatment group, ###, P<0.0001 vs. IL-1β+ GW9508 group, n=4-5).

Activation of SOX9 is recognized as a key event in the synthesis of type II collagen and aggrecan. The results in Figure 5 show that while IL-1β reduced the expression of SOX9 by roughly half at both the mRNA and protein levels, treatment with 50 μM GW9508 almost completely restored SOX9 expression. To deduce whether CREB signaling plays a role in GW9508-mediated rescue of SOX9 expression, we assessed the effects of GW9508 on the phosphorylation of CREB. The results in Figure 6A show that agonism of GPR120 rescued the level of phosphorylated CREB to near baseline, which was reduced by IL-1β by nearly half. To further confirm the involvement of CREB, we employed the selective protein kinase A inhibitor H89. As shown in Figures 6B, 6C, inhibition of CREB completely abolished the effects of GW9508 on SOX9 expression and even resulted in a 10% greater reduction in SOX9 expression at both the mRNA and protein levels. Thus, GW9508-mediated rescue of SOX9 expression is mediated through CREB signaling. Finally, we verified the involvement of GPR120 in GW9508-mediated increased SOX9 expression using the GPR120 antagonist AH7614. As shown in Figure 7, antagonism of GPR120 completely abolished the effects of GW9508 on SOX9 expression at the mRNA and protein levels.

**Figure 8:**
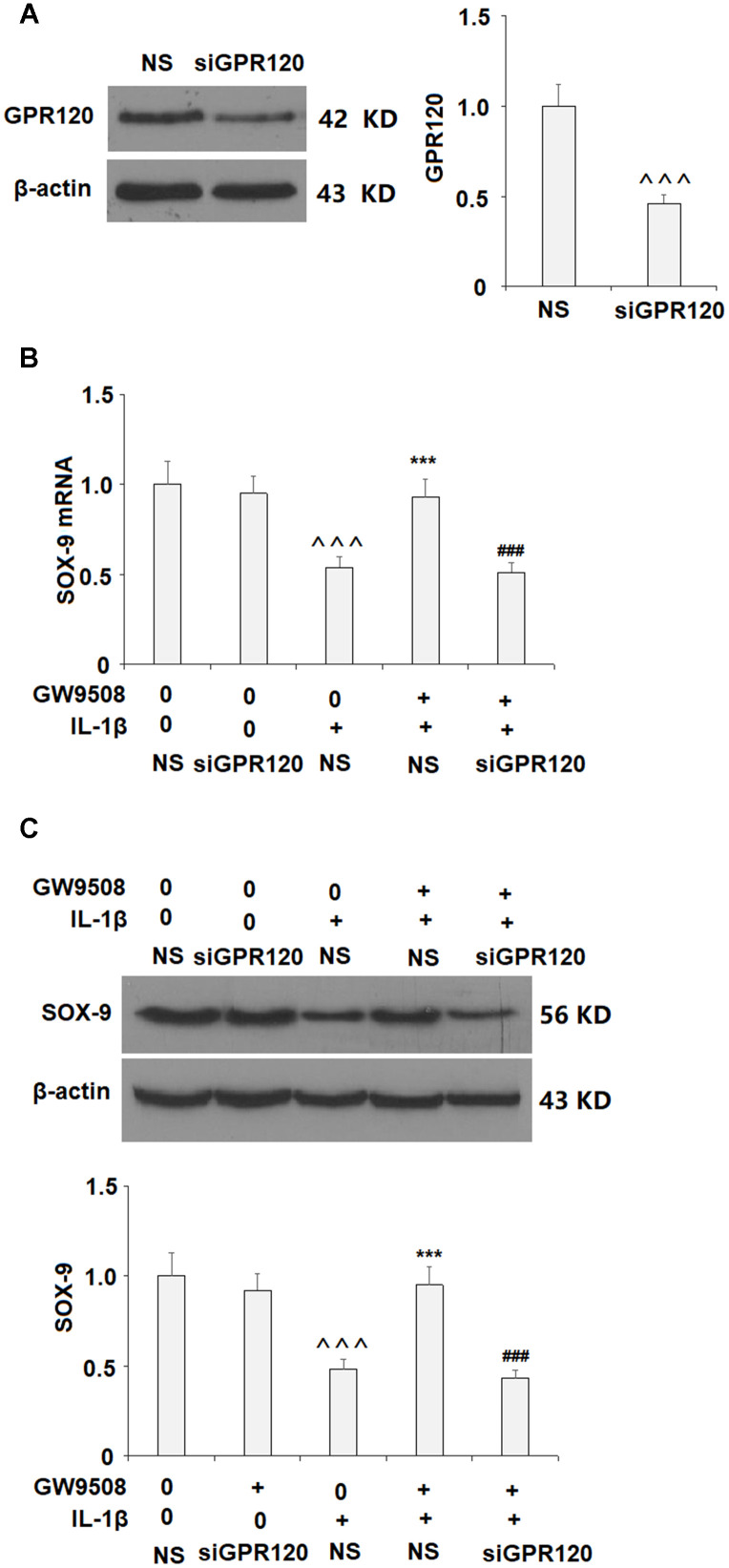
**Knockdown of GPR120 abolished the protective effects of GW9508 on the expression of SOX-9.** Cells were transfected with non-specific siRNA (NS) or GPR120 siRNA (siGPR120) for 24 h, followed by stimulation with or without GW9508 (50 μM) for 24 h. (**A**) Western blot analysis revealed the successful knockdown of GPR120. (**B**) SOX-9 mRNA; (**C**) SOX-9 protein (^^^, P<0.0001 vs. control group; ***, P< 0.0001 vs. IL-1β treatment group, ###, P<0.0001 vs. IL-1β+ GW9508, n=4-5).

The expression of GPR120 was then silenced by transfection with GPR120 siRNA to clarify whether GPR120 is involved. Successful knockdown of GPR120 is shown in Figure 8A. Importantly, knockdown of GPR120 abolished the effects of GW9508 on SOX-9 expression (Figure 8B and Figure 8C). These findings suggest that GPR120 is indeed the receptor involved in GW9508-mediated increased expression of SOX-9.

### The GPR120 agonist TUG891 prevented IL-1β-induced damage in chondrocytes

It should be noted that GW9508 is an agonist for not only GPR120 but also GPR40. To further elucidate the effects of GPR120 against IL-1β-induced damage in chondrocytes, another GPR120 agonist TUG891 was used. Consistently, we found that TUG891 significantly prevented IL-1β-induced reduction of type II collagen and aggrecan at both the mRNA and protein levels (Supplementary Figure 1). Notably, treatment with TUG891 prevented IL-1β-induced reduced SOX-9 expression (Supplementary Figure 2).

### Agonism of GPR120 prevented IL-1β-induced activation of NF-κB

Finally, we set out to explore the molecular mechanism through which GW9508 could influence IL-1-induced stimulation of IL-6 and IL-8 production. The transcriptional factor NF-κB has been shown to play an important role in regulating the expression of pro-inflammatory cytokines, including IL-6 and IL-8. Here, we found that IL-1β stimulation induced about 2.8-fold accumulation of p65 in the nucleus, which was significantly prevented by GW9508 (Data not shown). Additionally, the results of luciferase activity assay demonstrate that exposure to IL-1β induced approximately 161-fold higher promoter activity than the control, which was reduced by the presence of GW9508 (Supplementary Figure 3). Importantly, the inhibitory effects of GW9508 on NF-κB activation were abolished by knockdown of GPR120, suggesting that the inhibitory effects of GW9508 on NF-κB activation are mediated by GPR120.

## DISCUSSION

The present study provides evidence of the involvement of GPR120 in mediating IL-1β-induced destruction of the articular extracellular matrix in ATDC5 chondrocytes. Recent research on the role of GPCRs in OA has yielded some noteworthy results. For example, a 2019 review reported that there are presently 92 different GPCRs known to be involved in bone function and disease, including GPR120. This includes GPR120-mediated bone remodeling in OA [21, 22]. Another study demonstrated the involvement of GPR120 activation in determining whether bone marrow mesenchymal stem cells differentiate into adipocytes or osteocytes [23]. Increased bone turnover and altered bone metabolism have long been recognized as contributing factors in OA [24]. However, the role of GPR120 in cartilage homeostasis is not well understood. We began our experiments by measuring the expression level of GPR120 in rat ATDC5 chondrocytes and found that GPR120 is indeed expressed in this cell type. *In vitro* stimulation with IL-1β is a popular method for simulating the effects of OA in chondrocytes [25]. Next, we confirmed that IL-1β reduced the expression of GPR120 at both the mRNA and protein levels. This finding corresponds with previous research showing that GPR120 is inhibited by IL-1β [26]. Thus, we hypothesized that IL-1β-mediated downregulation of GPR120 may play a role in the pathogenesis of OA.

As inflammation is a major contributor to the progression of joint destruction in OA, the inhibition of proinflammatory cytokines is an important target. Recent research has shown that agonism of GPR120 can reduce the expression of IL-6 and other proinflammatory cytokines including IL-8 [27, 28]. Increased levels of circulating IL-6 and elevated IL-8 levels are associated with the severity of knee OA [29, 30]. Here, we found that agonism of GPR120 by GW9508 could significantly reduce the expression of these two cytokines, thereby exerting an anti-inflammatory effect. As the main hallmark of OA, cartilage degradation was the focus of our research. We observed that GW9508 and TUG891 could remarkably restore the levels of type II collagen and aggrecan expression, which were significantly reduced by IL-1β. SOX9, which plays a central role in the synthesis of type II collagen and aggrecan [31]. Therefore, promoting the expression of SOX9 may be a valuable treatment strategy to slow or potentially reverse cartilage destruction. A 2019 study demonstrated that altered expression of SOX9 and runt-related transcription factor 2 was correlated with chondrocyte health, with SOX9 expression being reduced in OA chondrocytes [32]. Importantly, a contemporary study using IL-1β-induced primary human chondrocytes and an OA mouse model showed that overexpression of SOX9 could ameliorate the pathogenesis of OA both *in vitro* and *in vivo* [33]. Here, we investigated the effect of agonism of GPR120 on SOX9 expression in ATDC5 chondrocytes and found that GW9508 and TUG 891 significantly rescued IL-1β-induced reduced expression of SOX9. Remarkably, GW9508 almost completely restored SOX9 expression at both the mRNA and protein levels.

To elucidate the pathway involved in GPR120-mediated rescue of SOX-9, we assessed the involvement of the CREB pathway. The CREB pathway is known to play a role in OA by regulating the expression of degradative enzymes upon activation by IL-1β [34], but the mechanisms of CREB signaling are complex and incompletely understood. We found that inhibition of CREB signaling abolished the effects of GW9508 on SOX-9 expression, thereby demonstrating the necessity of the CREB pathway in GPR120-mediated SOX-9 overexpression. GPCRs activate heterotrimeric G proteins by ligand interactions and play important roles in various physiological processes. Activation of adenylyl cyclase (AC) by GPCRs via coupling to Gs stimulates cAMP-dependent PKA, one of several protein kinases that can phosphorylate CREB. Similar to GPR40, GPR120 is usually coupled with the Gq protein family [35]. Based on the findings of the current study, we speculate that the effects of GW9508 on SOX-9 expression might be mediated by GPR120 via coupling to Gs in chondrocytes. Further investigation will provide a complete picture of the underlying mechanism.

Finally, we employed the GPR120 antagonist AH7614 and knockdown of GPR120 to confirm that GPR120 is indeed required for the effects of GW9508 on SOX9 expression. While GW9508 almost completely restored SOX9 expression, the addition of GPR120 antagonist and knockdown of GPR120 abolished this effect. Thus, GPR120 is the target through which GW9508 exerts its protective effects against OA. In conclusion, we first show that GPR120 is expressed in ATDC5 chondrocytes and is downregulated by IL-1β. Secondly, we show that agonist of GPR120 by GW9508 confers an anti-inflammatory effect by reducing the IL-1β-induced expression of IL-6 and IL-8. Importantly, we found that agonism of GPR120 by GW9508 prevented IL-1β-induced activation of NF-κB, suggesting that blockage of NF-κB might participate in mediating the protective effects of GW9508 against IL-1β-induced expression of IL-6 and IL-8. Third, GW9508 was able to prevent IL-1β-induced reduced type II collagen and aggrecan levels, which is likely via increased expression of SOX9. Finally, we show that GW9508-mediated rescue of SOX9 is reliant on the CREB pathway and that GPR120 is indeed required for these results. Further research will allow us to explore other pathways through which GPR120 might affect chondrocyte function.

A major limitation of this study is that the molecular mechanisms whereby agonism of GPR120 suppresses IL-1β-induced reduction of mRNA levels of type II collagen and aggrecan still need to be elucidated in future studies. The second limitation is that only *in vitro* experiments with IL-1β-stimulated human articular chondrocyte cell models were performed in this study. The pathological mechanism of OA is complex [36]. Various risk factors have been shown to be involved in the progression of OA [37]. In addition to IL-1β, other inflammatory cytokines and chemokines have been reported to play important roles in the development of OA [38]. Additionally, other types of cells, including macrophages, osteoclasts, and osteoblasts, are also involved. Therefore, *in vivo* experiments with animal models will be helpful to confirm the pharmacological function of GPR120 in OA.

## MATERIALS AND METHODS

### Cell culture and treatment

ATDC5 chondrogenic cell line was purchased from Sciencell and cultured in a differentiation medium containing DMEM/F-12 (1:1) with GlutaMAX I (Gibco, USA) supplemented with 5% FBS (Gibco, USA), 1% sodium pyruvate, and 0.5% gentamicin (Gibco, USA) for 1 week prior to experimentation. MIN6 cells were stored in a humidified incubator containing 5% CO_2_ at 37 °C. The culture media was replaced with fresh media every second or third day. To test the expression of GPR120, the cells were exposed to 10 ng/ml IL-1β for 12, 24, and 48 h. For subsequent experiments, the cells were exposed to 10 ng/ml IL-1β (R&D systems, USA) in the presence or absence of 50 μM GW9508 (Cayman Chemical, USA) or 10 μM TUG891 (Cayman Chemical, USA). H89 (10 μM) (Sigma-Aldrich, USA) was used in the CREB inhibition experiment, and AH7614 (1 μM) (Cayman Chemical, USA) was used in the GPR120 inhibition experiment.

### Real-time PCR

To detect the mRNA levels of GPR120, IL-6, IL-8, and SOX9, the total RNA was extracted from ATDC5 cells using an RNA Isolation Kit (Thermo Fisher scientific, USA). To generate cDNA, a total of 2 μg mRNA was subjected to reverse transcription PCR (RT-PCR) using an iScript cDNA Synthesis Kit (Bio-Rad, USA). Quantitative real-time PCR was performed using the SYBR Green MasterMix method and a LightCycler 96 Real-Time PCR System (Roche, Switzerland). The results are presented as normalized to glyceraldehyde-3-phosphate dehydrogenase (GAPDH) using the 2^-ΔΔCt^ method. Information on primer sequences is shown in Table 1.

**Table 1:** The primers sequences.

**Target gene**	**Upstream Sequence (5’-3’)**	**Downstream Sequence (5’-3’)**
**GPR120**	TGGAGATGCACATTGTTTGGAGA	AGCCTCCAAGTGGTGGAGTGA
**SOX-9**	GACGTGCAAGCTGGGAAAGT	CGGCAGGTATTGGTCAAACTC
**Collagen 2**	CGCCACGGTCCTACAATGTC	GTCACCTCTGGGTCCTTGTTCAC
**Aggrecan**	TCTC TGGCATTGAGGACAGCGAAG	TCCAGTGTGTAGCGTGTGGAAATAG
**IL-6**	CGG- GGTACATCCTCGACGGCATCT	GTGCCTCTTTGCTGCTTTCAC
**IL-8**	TTTCTGTTAAATCTGGCAACCCTAGT	ATAAAGGAGAAACCAAGGCACAGT
**GAPDH**	ACCCCTTCATTGACCTCAAC	CTTGACGGTGCCATGGAATT

### Western blot analysis

The expression levels of GPR120, IL-6, IL-8, and SOX9 were assessed via western blot analysis. Briefly, proteins were extracted from ATDC5 chondrocytes using cell lysis buffer (Cell Signaling Technologies, USA) supplemented with protease inhibitor cocktail. Following centrifugation at 10000 × g, the supernatant protein was collected from the samples. A BCA protein concentration assay was used to assess the protein concentrations in accordance with the manufacturer’s manual (Sigma-Aldrich, USA). Then, 20 μg protein was loaded onto an 8-12% SDS-PAGE gel for electrophoresis and transferred onto a PVDF membrane (Bio-Rad, USA). To block the nonspecific binding sites, the samples were incubated with 5% non-fat milk in TBST, followed by primary antibodies overnight at 4 °C, and HRP-conjugated secondary antibody at room temperature (RT) for 1 h. The protein bands were then visualized via ECL chemiluminescence (Thermo Scientific, USA). The resulting optical density was quantified using Image J software (NIH, USA). The following antibodies were used in this study: GPR120 (1:1000, #ab230869, Abcam, USA); type II collagen (1: 500, #MAB8887, Chemicon, USA); aggrecan (1: 500, #ab3778, Abcam, USA); SOX-9 (1:1000, #ab59252, Abcam, USA); β-actin (1: 10000, #ab8227, Abcam, USA); NF-κB p65 (1:1000, #ab16502, Abcam, USA); Lamin B1 (1: 5000, ab65986, Abcam, USA).

### ELISA

After the indicated treatment, the culture media was collected for centrifugation at 1000 × g. Commercial ELISA kits were used to measure the expression levels of IL-6 and IL-8: human IL-6 ELISA Kit (#D6050, R&D Systems, USA); human IL-8 ELISA Kit (#D8000C, R&D Systems, USA). Cell lysates were obtained by lysing the cells using cell lysis buffer. The supernatant was then collected, and the protein secretion levels were assayed using the ELISA kits in accordance with the manufacturer’s instructions. The resulting data are presented as fold changes.

### Measurement of luciferase activity

NF-κB activity was examined using a luciferase reporter assay*.* Cells were co-transfected with GPR120 siRNA, an NF-κB promoter firefly luciferase plasmid (CloneTech, USA), and a controlled renilla luciferase plasmid. At 24 h post-transfection, the cells were stimulated with IL-1β (10 ng/ml) with or without GW9508 (50 μM) for 24 h. The total cell lysates were collected to determine the dual luciferase activity of renila and firefly luciferase using a luciferase dual assay kit (Promega, USA).

### Statistical analysis

The experimental data in the present study are expressed as means ± SD. Statistical differences between groups were compared using one-way ANOVA, followed by Turkey's test. All experiments were completed in triplicate. A P-value (p) of less than 0.05 was considered to be a significant difference.

## Supplementary Material

Supplementary Figures
