# Supplementary material for: Agonism of GPR120 prevented IL-1β-induced reduction of extracellular matrix through SOX-9
Source: Aging (Albany NY). 2020 Jun 24;12(12):12074–85. doi: 10.18632/aging.103375 (PMC7343462; doi:10.18632/aging.103375)
Supplement: Supplementary Figures [file aging-12-103375-s001..pdf]

SUPPLEMENTARY FIGURES

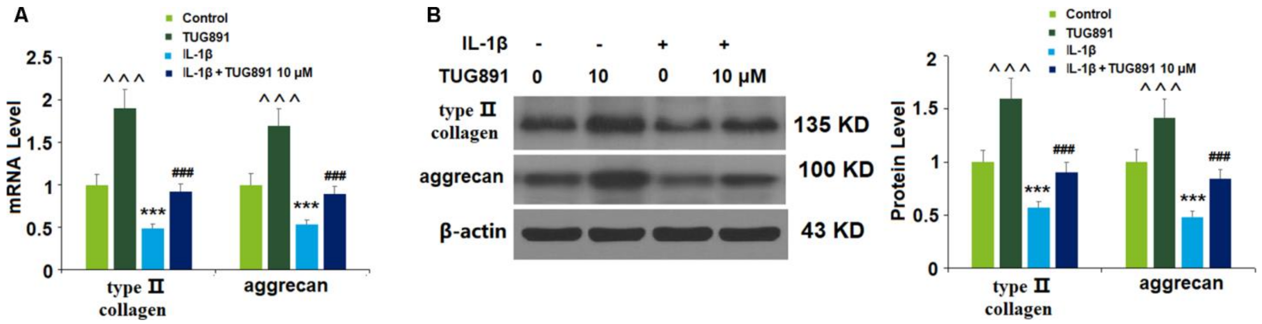

Supplementary Figure 1. Treatment with TUG891 prevented IL-1β-induced reduction of type II collagen and aggrecan in ATDC5 chondrocytes. Cells were treated with IL-1β (10 ng/ml) with or without TUG891 (10 μM) for 24 h. (**A**) mRNA of type II collagen and aggrecan; (**B**) Protein of type II collagen and aggrecan (^^^, ***, P<0.0001 vs. control group; ###, P<0.0001 vs. IL-1β treatment group, n=4-5).

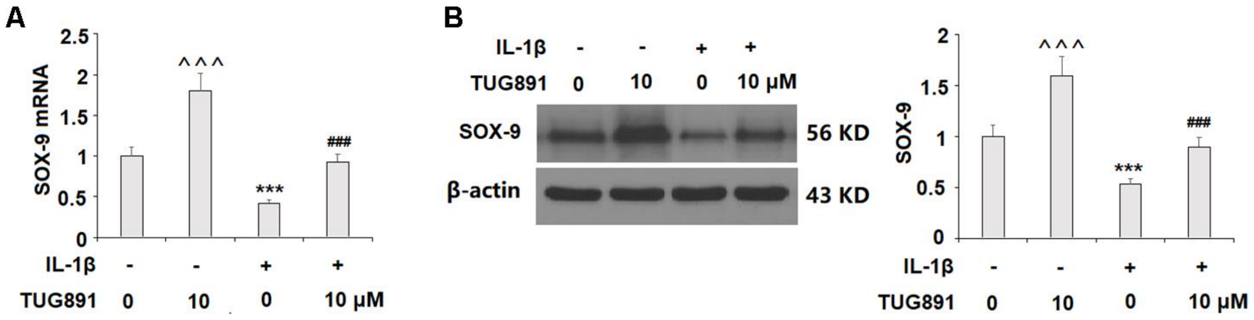

Supplementary Figure 2. Treatment with TUG891 prevented IL-1β-induced reduction of SOX-9 in ATDC5 chondrocytes. Cells were treated with IL-1β (10 ng/ml) with or without TUG891 (10 μM) for 24 h. (**A**) mRNA of SOX-9; (**B**) Protein of SOX-9 (^^^, ***, P<0.0001 vs. control group; ####, P<0.0001 vs. IL-1β treatment group, n=4-5).

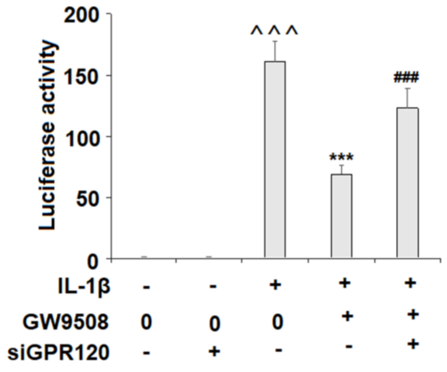

Supplementary Figure 3. GW9508 suppressed the activation of NF-κB. ATDC5 chondrocytes were transfected with GPR120 siRNA for 24 h, followed by stimulation with IL-1β (10 ng/ml) with or without GW9508 (50 μM) for 24 h. (**A**) Levels of nuclear NF-κB p65 were measured; (**B**) Luciferase activity of NF-κB promoter was measured (^^^, P<0.0001 vs. control group; ***, P<0.0001 vs. IL-1β treatment group, ###, P<0.0001 vs. IL-1β+ GW9508 treatment, n=4-5).
